# (Dis)advantaged positions in accessing gender-affirming healthcare in Finland: an intersectional qualitative study of foreign-origin transgender people

**DOI:** 10.1186/s12913-022-08654-3

**Published:** 2022-10-25

**Authors:** Mercédesz Czimbalmos, Shadia Rask

**Affiliations:** The Finnish Institute for Health and Welfare (THL), Helsinki, Finland

**Keywords:** Intersectionality, Transgender health, Gender-affirming care, Healthcare, Migration

## Abstract

**Background:**

An increasing body of scholarship focuses on transgender individuals’ experiences when accessing gender-affirming healthcare. However, the experiences of transgender individuals who identify as being of foreign-origin in Finland have rarely been studied. This study aims to fill the gap in research and contribute to the understanding of the experiences of transgender individuals who also identify as belonging to the foreign-origin populations in Finland.

**Methods:**

Fourteen semi-structured qualitative interviews were conducted and analyzed with reflexive thematic analysis (RTA), through the framework of intersectionality. The interviews were part of a broader sample of qualitative data, collected about the experiences of sexual and gender minorities among the foreign-origin populations in Finland.

**Results:**

The analysis showed two main interconnected themes. Firstly, perceived barriers when accessing gender-affirming care. In this theme, the intersections of transgender identity, foreign background, class, and age affected the experiences of the individuals. Secondly, the necessity of “performing identities:” the intersections of class, transgender identity, nativity, and race affected those.

**Conclusion:**

The findings of the current study suggest that the intersectional aspects of individual identities create structural inequalities in the Finnish gender-affirming healthcare system. To tackle these inequalities, further research is needed on the healthcare experiences of gender minorities in Finland both within and outside the scope of transgender-specific healthcare.

## Introduction

The current “trans law”^1^ [1] of Finland allows for Finnish citizens, and permanent residents in Finland to receive gender-affirming care. During the past years, transgender rights in the country have been at the forefront of both national and, to an extent, international discussions. The current law has widely been discussed and even criticized by academic and non-academic circles alike 2 [2, 3]. Much of these discussions are connected to the compulsory sterilization as a prerequisite for legal gender recognition stated in the law, and to the currently practiced gender-affirming processes. These practices follow the ICD^3^-10 diagnostic codes as opposed to those of the ICD-11, which —as elaborated later— do not pathologize transgender identities.^4^ Previous research shows that when the role of mental health professionals in the transitioning process is merely diagnostic, and not solely supportive, the mental health professionals are often viewed as gatekeepers by transgender patients, resulting in negative effects on the patients’ experiences with healthcare providers [5, 6]. Discussions around similar approaches have long addressed concerns and rejected approaches that tend to focus on ascertaining whether a patient is “trans enough.” Scholarly research has shown negative impacts of the pathologization of transgender identities, [7] and lights the path to new approaches. These approaches focus on the presence of gender dysphoria and on informing the patients on the potential risks and benefits of particular gender affirmation treatments —leaving the decisions in the hands of the patients [8]. Transgender individuals are more prone to experiencing depression, anxiety, or loneliness [9]. Such experiences have previously been associated with e.g., discrimination in housing or employment, physical and verbal abuse, social harassment related to gender presentation, or the lack of gender-affirming treatment [10]. The prevalence of these conditions among this group exceeds that of the general population. These psychological problems tend to decrease after the transition process and after receiving gender-affirming care [9], and the life quality of the individuals concerned tends to increase [11]. Gender-affirming care, however, is not equally accessible to all. In the United Kingdom, for example, high rates of dissatisfaction were reported concerning the access to gender identity clinics due to the long waiting lines, the small number of clinics and non-binary individuals often facing specific issues in accessing care [5, 12]. Moreover, healthcare professionals often have a limited understanding of pathways and clinical practices in the holistic treatment attempts of transgender patients [13].

Due to the recent violations against the fundamental rights of sexual and gender minorities both in- and outside the European borders, the number of individuals who are (contemplating on) relocating to Finland may potentially be growing. As such, the number of transgender individuals from foreign backgrounds may also be significantly higher in the foreseeable future. In order to offer better (gender-affirming) health services for individuals from such backgrounds, it is especially important to understand the shortcomings of the current system.

The aim of this study is to fill a gap in existing research and analyze the experiences and perceptions of gender-affirming care in Finland among those who identify as belonging to the foreign-origin populations, via an intersectional approach. In this article, the terms *transgender* and *trans* are used to describe identities of individuals, whose gender identity does not conform to the gender label assigned to them at birth. Trans people may identify with a gender identity that is different from their assigned gender, may identify as non-binary, genderfluid, or resist any categorization [14, 15]. Among the informants of the current study are transgender men, women, and non-binary informants alike. The authors of the study acknowledge the fluid nature of these identities. The term “foreign-origin” (fi. *ulkomaalaistaustainen)* refers to both those individuals, who immigrated to Finland or those, who were born and raised in Finland due to their ancestors, self-identify as being of foreign-origin. The authors of this study would like to emphasize that by using the term “foreign-origin,” they do not imply or make assumptions of individuals’ identity and are considerate of the fact that not all individuals from migrant or postmigrant backgrounds identify themselves as “foreign-origin.” The phrase was chosen due to the diversity of the informants of the study, and due to the self-identification of the informants.

The present article seeks to answer the research questions: *What are the main challenges foreign-origin transgender people face in accessing gender-affirming care in Finland?* and *What changes need to be implemented to improve access to healthcare, and thus the quality of life, of these individuals?*

### Gender affirmation treatment in Finland and the ICD-10 diagnostic codes

Currently, gender-affirming care in public healthcare is legally administered in Finland by psychiatric-led work groups in the two outpatient clinics, trans polyclinics —often referred to as “transpoli” or “trans clinic”— in Helsinki and Tampere [16, 17]. The clinics utilize the diagnostic codes of the ICD-10. After receiving a diagnosis from either of these clinics, the gender-affirming treatment of the patient is administered along a personalized path and may entail hormone treatment or certain surgical procedures [16–18]. Due to the current legislation, if a trans person wants to correct their legal gender marker, they are required to undergo sterilization process. In addition, there is normative pressure on trans people to undergo hormone therapy and/or surgical procedures even if they would prefer not to [9]. Several international studies argue that in many countries trans identities and experiences are still pathologized and that requirements for legal gender recognition (such as sterilization and divorce) do not fit the human rights standards [19, 20]. Along similar lines, the forthcoming World Professional Association for Transgender Health Standards of Care [21] includes new guidelines on trans-specific care, and explicitly uses the term transgender and gender diverse (TGD), whilst specifically reflecting on both individuals with or without treatment requests for transition-supportive treatments (e.g., non-binary people). This means that this version will both recognize gender diverse individuals, and that not all individuals hope to go through transition processes. Discussions around the problematic nature of the ICD-10 diagnostic codes and the necessity of developing trans-specific care have also reached Finland. They are mainly – but not solely – connected to the issues centering around the ICD-10 protocol, instead of the ICD-11, or more precisely to the Informed Consent Model. By changing the legislation to the ICD-11 model, the psychotherapy/gatekeeping requirement from the diagnostic process would be removed [8]. Shifting the diagnostic model towards that of the ICD-11 would, in practice, mean that the position of the mental health professionals in the transition process would be changed to a non-mandatory involvement, from the diagnostic role. A person therefore would be able to access trans-specific healthcare after a self-diagnosis assisted by a primary healthcare provider, who would then coordinate the access to the care based on informed consent [22].

## Methods

### Data collection and selection

The data for this study comprises semi-structured qualitative interviews (*n* = 14) conducted with individuals who are of trans experience and self-identify as members of the foreign-origin population in Finland.

The data collection took place within the Manifold More project of the Finnish Institute for Health and Welfare (THL) with a participatory approach. During the research process, the researchers built up a partnership with various projects and organizations who work directly or indirectly with sexual and/or gender minorities in the Finnish society. The study design and data collection drew on close collaboration with grassroot experts, in line with the practices of participatory research. The key grassroot experts collaborated with the project in their professional role and working hours. Crucial parts of the collaboration included suggesting participants for the study and disseminating information about the study in their own networks. Drawing on the participatory approach, the researcher was able to reflect on and address issues that may not have been visible through traditional research methodologies. The selected interviews are part of a larger sample of qualitative data focusing on sexual and gender minorities who self-identify as members of the foreign-origin population in Finland. In addition to the data used in this study, the larger sample entails eight focus group interviews with the representatives of the projects and organizations who were of support in the data collection and the knowledge creation, and additional twenty-six semi-structured qualitative interviews with individuals who self-identify as belonging to sexual and/or gender minorities and to the foreign-origin population in Finland. The individual informants for the broader research were recruited via an open call distributed online on the website of THL, and via the organizations and projects who were present at the focus group interviews. Moreover, snowball sampling was also applied. The interviews were conducted between February and July 2021.

The inclusion criteria for the data collection were that the individuals were over 18 years, and self-identified as members of the target population. The focus group interviews were used when constructing the interview outline for individual interviews. Questions during the individual interviews reflected on the backgrounds of the informants, on their self-perceived identities, and on the daily challenges they face in the Finnish society. As such, questions such as “When did you (or your family) immigrate to Finland?” or “What are the day-to-day challenges you experience as a [insertion based on self-definition] in Finland?” were asked from the informants. Interviews analyzed in this particular study were conducted in English, French, and Arabic.

When conducting the interviews, the researcher strove to ensure inclusive data collection by carrying out the data collection flexibly in locations and time frames which suited the informants the most, including outside of office hours, for example over the weekend. The researcher also considered the informants’ preferences concerning the usage of language, and in case the informants hoped to proceed with the interviews with a specific interpreter, this was ensured. Informed consent in oral form was obtained from the participants of the study —along the suggestions of the Ethics Committee of THL— to protect their identity. All interviews were audio-recorded and transcribed verbatim in full. After transcribing the interviews, the audio records were deleted. In the current study, interviews of informants who talked about gender-affirming healthcare in Finland and who were not questioning their gender identities at the time of our interviews were analyzed.

### Informants

The youngest informant was in their 20 s and the oldest in their 60 s. The informants came from diverse backgrounds, from countries in Europe, Eurasia, Africa, North and South America. The sample included both voluntary and involuntary migrants and individuals who were born in Finland but self-identified as belonging to the foreign-origin population in Finland. Due to the small size of this segment of the population, the authors opted to omit certain information from the article, in order not to reveal the identity of the informants. For the same reason, informants are anonymized throughout the study. They are named with one-letter abbreviations (e.g., “A” or “B”) and are categorized in “age groups” (under 35, above 35) (Table 1). These abbreviations were consciously chosen to avoid using culturally insensitive or gendered names. For easier readability of interview extracts, they were edited, and unnecessary linking words were removed from them.

**Table 1:** Overview of the age groups and identities of the informants

Abbreviation	Age group	Identity
A	under 35	trans man
B	under 35	trans woman
C	over 35	trans woman
D	over 35	trans man
E	over 35	trans man
F	under 35	transmasculine, non-binary
G	under 35	transmasculine, non-binary
H	under 35	trans woman
I	under 35	trans woman
J	under 35	trans man
K	over 35	trans woman
L	under 35	trans woman
M	under 35	transfeminine, non-binary
O	under 35	transmasculine, non-binary

### Positionality of the authors

The authors reflected on their positionality particularly in terms of the (power) relations between the researcher and the research participants, as well as in the chosen theoretical and methodological choices. The authors recognized the power imbalance between the researcher and the researched parties – in particular, the participants of the individual interviews, many of whom were in vulnerable positions due to the intersections of their multiple (marginalized) identities. Many of the participants voiced willingness and comfortability to participate in the study as they knew that the researcher responsible for the data collection is a member of multiple minority groups, and that the broader project team members who have direct access to the material also includes individuals of minority backgrounds.

During the research process, the researcher strove to apply constant reflexivity. This in practice meant e.g., that the researcher responsible for the data collection constantly examined her self-positioning in relation to the researched population. To balance the biases of the authors applied constant self-reflexivity during the analytical and writing process. The research group also discussed choices related to the data collection and questions related to the interview situations.

The key grassroot experts collaborated with the project in their professional role and working hours. While a collegial relationship existed between the researchers and the collaborating experts, the resources of collaborating NGOs, especially in terms of financial resources, are often scarce and their projects are often temporary. These dimensions were openly discussed in the research process, and all parties felt that the collaboration and research spoke for the work that the NGOs and individual projects do to support different groups within sexual and gender minorities, hopefully helping to secure better finances in the future.

The authors recognize that this work would not have been possible without the collaboration of all the different organizations and their representatives, who provided their input and expertise.

and help throughout the different steps of the project. The authors have sought to use the power and privilege related to their position in a governmental research institute to give voice to the individuals who participated in the interview and shared their lived experiences.

### Theoretical framework and analytical approach

#### Intersectionality

Intersectionality [23] is a framework that allows for an understanding of how multiple intersecting systems of privilege and oppression operate at the macro level of society to influence the everyday lives of individuals at a micro level [24–26]. While there are several different scholarly approaches to intersectionality, three elements are perhaps common to each of these: individuals are assumed to have multiple intersecting identities; a dimension of power and oppression is within each identity; these identities of individuals are created by the socio-cultural context and as such they are mutable [27]. Intersectionality has the potential of highlighting the interaction of various social identities, in various contexts [28] and as such is a useful tool when uncovering the workings of the multiple systems of inequality in unpacking the social determinants of health [29]. Intersectionality resists the additive approach to social categories, such as gender, class, race, or sexuality, rather it focuses on “what is created and experienced at the intersection of two or more axes of oppression” [30]. Research based on intersectional approaches holds the potential of facilitating the development of structural-level interventions [25]. Solely examining interactive effects of social categories is insufficient to qualify as an intersectional approach, however [27, 31]. Along the lines and suggestions of previous studies [27] the researchers opted to examine the effects of these social categories on the lives of the individuals by examining how power and inequality play a role in their lives, acknowledging the fluidity of these categories and their significance.

To be able to shed light on possible structural inequalities in various systems, the interview outline of the collected qualitative material was created by taking the framework of intersectionality into consideration, and via reflecting on the everyday experiences of the individuals in relation to their self-described identities—as Lisa Bowleg [24] suggests. In the current study, intersectionality was not only considered in the analytical phases, but also when designing the study, collecting the data.

#### Analytical approach

The analytical approach employed in the current study is reflexive thematic analysis (RTA) [32]. RTA is a flexible analytical method that is suitable when seeking answers to a wide range of questions, connected to e.g., experiences, understanding, or perceptions [33]. It has the potential to create comprehensive narratives in the analytical process [34]. In RTA, themes reflect a pattern of shared meaning, organized around a central organizing concept, or idea [35]. As a first step of the analytical process, the interview transcripts were read and re-read several times. In the following step, the material was manually coded, then re-coded by the first author. Then, the codes were organized into broader candidate themes which were identified through the intersectionality-driven deductive analytical process. After these steps, the authors discussed the themes together until reaching a consensus on the theme development. As a result of the process, two main themes were developed from the material: *perceived barriers when accessing gender-affirming healthcare*, and the *necessity of “performing identities.”*

## Results

Perceived barriers when accessing gender-affirming care: the intersections of transgender identity, foreign background, socioeconomic status, and age.

The most prevalent theme in the interviews centered around the range of barriers to receiving gender-affirming healthcare. Barriers when accessing different healthcare services cannot solely be identified as financial barriers, or as barriers pertaining to the availability of healthcare services in theory—while undeniably, these matters were also present in the narratives of the informants.

The first barrier many who did not speak Finnish^5^ sufficiently encountered was a linguistic one: finding adequate information on the processes proved to be difficult, resulting in informants relying on help from their close social circles. This barrier was prevalent in the entire process of seeking gender-affirming healthcare: when seeking information about the possibilities of receiving gender-affirming care, later when receiving calls for doctor’s visits and during the visits when discussing various matters with the medical professional. Admittedly, however, native English speakers or those who spoke English in a sufficiently high level were in a slightly better position than those who did not, as detailed later.

When passing this first barrier, and becoming familiar with the process, the second barrier encountered proved to be the one with the “gatekeepers”—as some informants called them. Much of this gatekeeper experience was connected to the step of acquiring a referral to the trans polyclinic, and to the actual “diagnostic process” —to the utilization of ICD-10— itself. This is well exemplified by the case of a trans man, A, who already had his gender marker changed in his passport and identification documents that were issued by the authorities of his country of origin. He has been accessing hormone treatment illegally for some time and recalled that when he arrived at the general practitioner (GP) to get his referral to the trans polyclinic, the GP asked a number of questions and performed an examination on him, which made him feel uncomfortable.

[…] he was like “When did you first know…” and “Why do you think you are a man?” and all of these, just ridiculous questions. I was just fuming, but I really wanted to get to transpoli, so I was just answering and just like really...biting my tongue […] he was getting very impatient, and then he’s done like a check upon me that I didn’t want. A physical checkup. […] He was like lifting up my jeans, and looking at my leg, and feeling my pulse from my feet […] I really felt like he didn’t have a clue, and he wanted to “perform doctor,” but he could have just...listened to me, and I went there informed of course, but they don’t believe that. So, that was really uncomfortable, and I felt sort of…vulnerable.

(A, under 35)

Among the most vulnerable informants were transgender asylum-seekers and refugees. They often described that they did not know much about their possibilities of receiving gender-affirming healthcare in Finland—partially due to the linguistic barrier, and partially due to the fact that the personnel they interacted with was not necessarily aware of matters connected to gender diversity and expression. A trans woman, B, who arrived in Finland as a quota refugee^6^ recalled, her social worker was not only unfamiliar with the processes of receiving gender-affirming healthcare but also questioned her womanhood:

My social worker told me: “You are a full woman. If I didn’t see it in the papers that you are a trans woman, I would have never guessed. I would think that you are a real woman.”

(B, under 35)

Other informants also suggested that employees of these supporting systems often lack not only trans-specific information, but an intersectional understanding of the needs of sexual and gender minorities, and even cultural competence altogether. Due to these shortcomings and the barriers resulting from them, individuals often inquired information from non-governmental organizations^7^ —that is if they were aware of their existence.

Even those, who have lived in Finland throughout their lives, or who have already spent a significant amount of time in the country before wanting to access gender-affirming care talked about having similar experiences. As many of them mentioned, they needed to visit multiple doctors until receiving the first referral to the trans polyclinic. A trans man, D recalled:

First, I went to a doctor who could give me the referral. Actually, the first doctor didn’t know what to do, so he sent me to a gynecologist, because obviously [says sarcastically]: that’s where you will deal with it!

(D, over 35)

It was not infrequent that informants who immigrated to Finland later in their lives have already received —medically informed or not— gender-affirming healthcare abroad. In these cases, the intersection of migration status and transgender identity caused power asymmetries between those who were able to access gender affirmation healthcare elsewhere and those who were not. C, a trans woman who moved to Finland as an adult had already been administered gender-affirming treatments for years and had a female gender marker on all her legal documents. She only decided to turn to the trans clinic due to the suggestion of her partner to —as she said— “become legible in the Finnish system.”

I was not particularly interested in interacting with the gatekeeper – on gatekeeper terms. So, I didn’t. I put on the Miyake suit^8^ and showed up, and it was like “Okay, well I am trans, I have been trans for a long time.” […] It was probably the weirdest and most hostile interview that the clinician ever had in that context. Because as far as I was concerned, I had all the power in that situation. I didn’t actually need them, and I was not interested in playing any of their games.

(C, over 35)

C received her Finnish diagnosis the same day. Later she told in the interview, that she is to be getting her bottom surgery and will rather pay for it abroad from her own finances because she “will not interact with the Finnish medical system around transitioning again.” Unlike C, a trans man, E, who has been living in Finland for over two decades initially paid his visit first to one of the Finnish trans polyclinics. Due to the lengthy processes, however, he decided to proceed with the transition process in his country of origin, another EU country. The lengthy processes from the inquiry of information to the actual accessing of the desired treatments proved to be one of the greatest perceived barriers not only for him, but for many others too. E, when wanting to be administered in the Finnish system with his existing diagnosis, was told he needs to go through the Finnish diagnostic process all over again. Similarly to C, he only wanted to “become legible” in the system. Having an existing diagnosis, however, allowed him to exercise some power and receive a diagnosis faster than it would have taken him otherwise.

[...] in the end, I kinda just pushed it a little bit […] I think they are not used to people talking back, because of course if you are a Finn, you are totally dependent on them, and you really have to obey and give all the answers and get all the ticks in the right boxes. But I had backup, so I think I could just be a bit...I was a bit of an ass, to be honest.

(E, over 35)

It is rather prevalent in the material that older informants tend to have slightly fewer difficulties when accessing the particular gender-affirming care they hope for. D reflected on this matter explicitly when saying he was “a bit older than some of the people he was being interviewed by.” The others did not talk about this specifically, but in comparison to the other informants, they seemed to have received their diagnosis —and thus, the first treatments— quicker than informants under thirty-five years of age.It is clear from e.g., E’s case, not (only) being a citizen or a resident of Finland can both be an asset, and a disadvantage: people who come from countries where it is possible to go through gender affirmation treatment may have the possibility to access treatment in their countries of origin. When talking about their medical transition process, F, a transmasculine non-binary informant under thirty-five, recalled that they “knew that they are not going to transpoli ever,” which was primarily connected to the experiences of their acquaintances with the process, who were “harassed, gatekept and just treated so poorly.” When getting into a secure socioeconomic status, F decided to proceed with their medical transition process abroad on their own expenses, in their country of origin within the EU where private practitioners may assist with certain aspects the medical transitioning process—while the general attitudes to (sexual) and gender minorities in the country is hostile. As they concluded: “it took like a lot of energy, and weird privileges and resources to navigate transitioning without transpoli in here.” The “weird privileges and resources” include those connected to the individuals’ financial, and socioeconomic status, and to their basis of migration. G, another trans-masculine non-binary informant under thirty-five, who was born outside of Finland, but had Finnish roots and essentially grew up in the country, reflected on similar privileges. Residing in Finland for longer —and, e.g., being older as well— may result in broader networks, and perhaps better established social and economic conditions too. When lacking these resources, privileges, and networks individuals often find themselves in particularly vulnerable positions.

As indicated above, trans refugees and asylum-seekers, who often arrive in Finland from countries that are particularly hostile towards sexual and gender minorities, described serious barriers when accessing certain treatments both in Finland and abroad. Their possibilities of accessing these in their countries of origin are, essentially non-existent. While waiting for their asylum-seeking claim to be processed, they are not entitled to gender-affirming healthcare in Finland, and of course, even after receiving their residence permit, they must go through the process of the trans polyclinics. H, a trans woman under 35 addressed this by saying “…the process started when I got my residence permit. And by starting, I don’t mean that I started to get hormones, I got my first hormone treatment at the end of 2018, and I arrived in 2015.” When receiving the residence permit takes longer, their access to treatment is delayed further, impacting both the psychological and the physical health of those concerned. I, another trans woman from asylum-seeking background told that she will “feel more comfortable when she will look like a woman.” J, a trans man summarized some of his concerns about his health due to the lack of gender-affirming treatment by saying:

I think that my body does not produce the right amount of hormones now, because I was on testosterone. I really need to be checked medically. […] I really need that medical support and I couldn't get it.

(J, under 35)

In theory, certain surgical procedures that may be part of the gender-affirming treatment are available in Finland in the private sector as well; individuals who have smaller incomes are hardly able to afford them. This naturally creates another, economic barrier for those who wish to access treatments in addition to those provided by the public healthcare system.

Lacking the possibility of accessing treatment in their countries of origin and lacking the financial means to accessing treatments in Finland, many of them opted to travel abroad. Without having their names, or gender markers changed in their personal identification this posed serious threats to them. Moreover, as suggested earlier, it was not uncommon for informants to access certain treatments —such as hormone therapy— illegally, without medical supervision, before having the diagnosis from the Finnish trans polyclinics. This was also common amongst those who came from asylum-seeking, or refugee backgrounds.

The necessity of “performing identities:” the intersection of class, transgender identity, nativity, and race.

Much of how one can perform their own identity and “prove their transgender identity” is dependent on various factors which are undoubtedly linked to their other identities and the intersections of those. Many informants felt the need to perform their gender and other identities in particular ways with confidence, to receive the treatments they sought to receive. D, who is a native English speaker, with an educational background in performing arts summarized:

Usually, when I go to the doctor’s I go in, and I go “I have this thing, but if you feel like fixing me, that would be great.” You know? Like I am always in awe of people who go into doctors’ offices and be like “I have this problem, can we fix it?” [….] in this situation [at the trans clinic] I was just like... “I know what I need, and I want to get it.” I never had a doubt that I was gonna go through. But I think it [being able to use his mother tongue] helped a lot […] I am a professional communicator, so...this I think also helps […] Everything that I do in my [professional] life, kind of trains me to be able to talk about these interior worlds.

(D, over 35)

Being able to express oneself in one’s own mother tongue can be a significant asset when accessing treatment—which has often been overtly and covertly suggested by not only native Finnish but also native English speakers.

Not having proficiency in certain languages and needing the help of interpreters may impact how individuals are able to access treatments—or other services, for that matter. Informants with asylum-seeking backgrounds who do not have sufficient language skills in English or Finnish, for example, have addressed that whereas they are provided an interpreter during their appointments at the clinics, when they receive correspondence or are in the need of filling out documentation before those, they often rely on favors, as they cannot afford translation services. B reflected on the matter as “If I ask someone with some paperwork, like some guy who lives here for long, they will ask for sex in exchange.”

M, a transfeminine non-binary informant briefly mentioned that they feel “more comfortable in English than the employees of the trans clinic do.” Their language proficiency had the potential of positively impacting their access to treatment. The experiences of K, a highly educated trans woman, point to the same supposedly positive impact. She was born and raised in Finland and has long been a member of international circles, where she became aware of various discussions around human rights approaches in gender-affirming care—already before visiting the trans clinic. She made sure that the clinicians were aware of both her knowledge in the field and her professional background. Her performance of class and her native Finnish skills contributed to her accessing gender-affirming healthcare significantly faster than many others could. As she noted, however, “not everyone is treated the same way” at the clinics.

I have done quite a heavy campaign for myself there. And they are a bit afraid of me, also, due to my [professional background]. So, of course, it feels bad that we should fight for equal rights. It should be automatic for anyone and everyone should be treated the same way.

(K, over 35)

Being aware of one’s privileges and having the ability to perform particular identities at the clinic was also present in C’s narrative, who, as above mentioned only wanted to “become legible” in the system. She was very conscious of the necessity of performing her identities during her one and only appointment at the trans clinic.

I can be very effective in using class privilege as a weapon when I need to. It’s a weapon I consciously learned to use […] the parts of this route in Finland are only possible, because I have immense amount of social privilege, and social and financial capital […] if I had the choice of using that privilege to perform a narrative that everyone should get to perform, I am going to do that, you know?

(C, over 35)

It is clear from the material that those who are unable to perform these identities, such as transgender refugees and asylum-seekers, are in particularly unprivileged and marginalized positions in the Finnish society and in the system of receiving gender-affirming care. Among the informants were a number of quota refugees, who were granted asylum in the country based on their gender identity and on the fact that they were targeted by serious human rights violations in their countries of origin. At the trans clinic, however, they found themselves in the position where they constantly needed to prove themselves to the professionals:

I didn’t come here, still looking like a man with a beard. I am a woman, in front of you, with everything. With all the surgeries and all the hair removed. And they are still asking me these questions again? […] I came here, I got no support I needed as a trans woman. I came through the United Nations as a woman to this country. I don’t get anything to help me.

(B, under 35)

L, another quota refugee described her experiences very similarly, addressing that while the workers of the trans clinic were never hostile to her, she felt that her situation as a refugee is not identical to that of a Finnish person:

They were kind of nice people, honestly. I think they kind of understood the point, but I guess they have some rules for Finns, and they use the same with us. I remember that when I was at the end of that “diagnostic process” I was shouting with the doctor during every meeting. I told many times that I didn’t come to Finland with a beard and a mustache, like a man. I came as a woman. I told them that already before [coming to Finland] I was using hormones. I didn’t go to them [the clinic] from zero and tell: “Now I feel like I am a woman and want to transition.” I was a woman from day one, and I needed the hormones from day one.

(L, under 35)

As a result of not receiving gender-affirming care in Finland for a long time, both L and B opted on accessing illegal hormone treatment and particular surgeries abroad, e.g., in Turkey, where they were familiar with the system, and were able to afford the treatments, even with the necessity to travel to the country. Despite their confidence in presenting their gender along the lines of the binary— which, as described later is suspected to be favored in the clinical process—, the class difference between them and other informants, their non-nativity, and the means through which they can perform their identities seemed to have delayed their access to treatment.

When it comes to performing one’s identity, it is also important to point out the rather normative perceptions of gender at the trans polyclinics. This was particularly dominant in the narratives of non-binary informants, who often thought that their non-binary genders may hinder their access to trans care. Moreover, informants who also identified as non-white felt that their racial identities could impact their access (or lack thereof) to treatments. M, a transfeminine non-binary informant for example described that despite them “being raised white, looking white, and having the white privilege” they are perceived as a person of color in Finland, which, in their own words is a “mindfuck.” Due to their country of origin, they often find themselves being associated with a strong, exaggerated sense of masculinity —which, being a transfeminine, non-binary person does not match their self-perception.

[…] what’s fucked up, is that at the transpoli as a non-binary person it’s way too difficult to get anything. Like anything! So, I did what a lot of people do: I am playing a binary role because that’s the way I found my way easier through the bureaucracy in the process […] I can talk to them [to the trans polyclinic] about the dysphoria. In my case, I am non-binary, but a lot of my dysphoria comes from looking too masculine and thought to be too masculine. You know, when it turns out that I am from [country of origin].

(M, under 35)

Those who identify themselves as people of color, or who are being perceived as people of color—and thus, lack the “white privilege” mentioned above by M—found it more difficult to make their identities understood when they are hoping to receive gender-affirming care. As a trans-masculine, non-binary informant addressed:

I am also nervous as a non-binary person, that I am “not binary-enough,” or I am not like, in their opinion “trans enough.” […] I just feel like the binary trans man is the thing that they would want me to go for, I would maybe say that I am a non-binary trans man or trans guy, but I don't fit that model […]. I have heard like...different kind of advice [from others]. For example, it’s better to lie that you are more binary than you actually are. […] and I am a bit nervous about how me being [racialized]...like...affects it. I don’t think they understand what things I say at times, like...I was talking about the “not being able to fit into any genders” […]

(O, under 35)

It was clear from the narratives of informants that particular ideas, biases are still present in the Finnish society about their countries of origin —in addition to those pertaining the normative ideas concerning gender—, which affected their experiences significantly, and often resulted in a stronger perceived necessity of performing their identities in particular ways that they deemed more comprehendible. This signals that there is a certain lack of cultural competence in not only the gender-affirming care setting, but perhaps also in the broader societal context, which may result in unequal treatment in general, and unequal access to certain treatments in particular in the Finnish society. It was indicated by most participants that they often drew certain conclusions not solely based on their own experiences but also based on information that they have acquired from their social circles in Finland, or previous patients at the trans polyclinics.

## Discussion

While the situation of gender minorities has undeniably developed in Finland over the past years, as the results of the current study show, trans persons still face several difficulties in the Finnish society. This is not unique to Finland of course, as previous research [37] has already addressed. In the Finnish context —as well as internationally— there are several changes to be implemented in order to enhance not only the quality of care of transgender individuals, but also their general position in the society.

One of the most prevalent examples that contributes to the inequalities transgender individuals experience in Finland is a result of the current “trans law” [1] and its shortcomings. These shortcomings are not only associated with barriers to gender-affirming care, and with the sterilization requirement, but also with the broader societal perception of gender minorities.As previous studies have pointed out, the concept of self-determination in gender-affirming care can be understood to be a key determinant of transgender health. Several international studies argue that the pathologization of trans identities and requirements for legal gender recognition (such as sterilization, and divorce) do not fit human rights standards [19, 20]. As previous research has already established, accessing gender-affirming healthcare has the potential of increasing the life-quality of transgender individuals, as it decreases gender dysphoria, and as such, has already been identified as a key social determinant of health among trans individuals [7, 11, 38]. Changing the “trans law,” and thus brining it more into line with higher international standards, and complying with human rights principles, the renewed law could potentially reduce the discrimination and stigma transgender individuals face on various realms in the society.

As trans people are often in the need of proving or performing their identities, or “need to prove their gender” to healthcare professionals to receive certain gender-affirming treatments [7, 13, 39]. In the Finnish context, the necessity of performing one’s identity is mostly, but not entirely, connected to the currently practiced diagnostic processes. This is well-exemplified with the cases of non-binary individuals, who often feel that they need to perform their gender in a particular way to receive gender-affirming care [13], who do not necessarily seek gender-affirming medical interventions. When they do, however, they might face the problem of how the normative culture of gender manifests and how it may shape their access to particular treatments in the transitioning process [6, 13, 40, 41]. This indicates that the culture of gender in which gendered bodies exist as a binary is still prevalent. Insisting on a strict binary is also often linked with heterosexuality as being the dominant driving strategy for the continuation of gender norms [42, 43], and with the very presence of transnormativity in the society. Transnormativity is a hegemonic ideology that structures transgender experience, narratives, and identification into a hierarchy of legitimacy that is dependent upon binary models and standards, regardless of whether the individual(s) wish to undertake medical transition processesor not [10]. These issues can stem from a lack of knowledge about gender diversity beyond the binary and can result in, e.g., barriers to healthcare or foregone care for non-binary patients [44, 45].

In addition to the inequalities stemming from the normative understandings of gender, this study also illustrated how nativity, citizenship status or language come into play in creating inequalities in different structures, as for example Kimberlé Crenshaw [46] has also illustrated previously. Matters of language and nativity were already present in the first steps of seeking information on gender-affirming care, as well as during the consultations with professionals of the trans polyclinics. These issues signal the lack of sufficient levels of cultural competence of healthcare professionals. Being a migrant or having ethnic minority status has previously been linked to unequal access to the health system, and cultural competence was indicated to be an efficient strategy of balancing health inequalities [47]. Nevertheless, the aspects of language and nativity, have received less attention in previous research than the intersections of race and class [29]. The findings of the study imply that in addition to increasing the competences of healthcare professionals connected to gender diversity and expression, competences connected to language and cultural matters are also to be focused on in order to provide more accessible and equal treatment from (trans) patients of diverse backgrounds.

Another matter that requires broader reflections in the Finnish context is connected to the centralized system of gender-affirming care in the country. Currently, gender-affirming care as such, is only administered in Finland in the two public outpatient clinics mentioned in the introduction, while accessing treatments that may be accounted as gender-affirming care, such as mastectomies, may be possible in certain private clinics. Regardless of the changes in the law, the issue of geographical burdens around the transitioning process must be taken into further consideration. The issue of geographical burdens when needing to access gender-affirming care has previously also been addressed by international research [13, 48, 49].

Due to the recent political atmosphere in several European countries (e.g., Hungary or Poland), and the increasingly hostile environments for transgender and gender diverse individuals, as well as sexual minorities, more detailed reflections on issues pertaining to the experiences and rights of sexual and gender minorities is of importance. In the Finnish case, these reflections are particularly crucial, as in the near future, the number of individuals who migrate to the country due to being prosecuted for sexual orientation, gender identity or expression (SOGIE) may be increasing. For similar reasons, it is important to reflect on the rights of transgender asylum seekers and quota refugees who may be granted residence permit in Finland due to SOGIE claims.

### Strengths and limitations

Previous research has not addressed the experiences of transgender individuals in Finland who also identify themselves as members of the foreign-origin population of the country. While the experiences of the foreign-origin populations of Finland have previously been studied, studies with the current foci have not been carried out in previous research.

Similarly, the research pertaining to health service experiences of transgender individuals in Finland has been scarce. This study therefore fills an obvious void in existing research, and carries significant implications for policy change, as well as future research to be carried out. In addition to the novelty of the topic of the study, a particular strength of it stems from the methods of data collection, and the application of the participatory approach and the inclusive data collection.

The main limitation of the study is that the informants included in the current sample are mainly located in close proximities of the two currently existing clinics where gender-affirming care is administered. As such, the experiences, and voices of those who face additional barriers connected to geographical proximities are not heard. A further limitation is that while the informants were of considerably diverse backgrounds, individuals from e.g., particular European countries that are hostile towards gender minorities are underrepresented in the sample.

## Conclusions

The present study provides an in-depth analysis of the experiences of fourteen individuals, who self-identify as transgender and as members of the foreign-origin population in Finland, when accessing, or when contemplating accessing gender-affirming healthcare in Finland.

Little scientific attention has been paid to this segment of the population. Therefore, this study fills a void in the existing research. The data analyzed in the study is part of a larger sample of qualitative material, collected via an intersectionality-driven [23–25] participatory approach. As such, it takes the knowledge and expertise of the grassroots experts into consideration. The goal of the study was to reflect on the experiences and perceptions of gender-affirming healthcare from an intersectional perspective.

The materials of the current study were analyzed vie reflexive thematic analysis [33]. The results of the analysis clearly indicate that trans individuals —of foreign-origin— face a number of barriers when wanting to access gender-affirming care in Finland. As a result of the RTA, two major themes that were identified in the sample. These were *perceived barriers when accessing gender-affirming care*, and the *necessity of “performing identities.”* The first theme was formulated around experiences of structural inequalities along the intersections of transgender identity, foreign background, class, and age. This theme mainly resulted from the currently applied ICD-10 diagnostic codes in Finland, but was also connected to matters concerning language skills, the countries of origin of the individuals concerned. The second theme was formulated along the intersection of class, socioeconomic status, transgender identity, nativity, and race, and was mainly connected to how individuals were (or were not) able to “perform” particular identities which may locate them in more privileged and powerful positions when hoping to access certain treatments.

As the results point out, depending on the intersecting aspects of the individuals’ identities, they may be in significantly advantaged or disadvantaged positions when accessing gender-affirming healthcare in Finland. In the current case, it is clear from the analyzed material that the informants perceived to have faced significant barriers and needed to pass several “gatekeepers” before being able to start the discussions with the trans polyclinics about their gender affirmation processes. Informants who had the means to access services elsewhere legally have been in an advantaged position compared to those, who did not have the possibility to do the same. Those who were aware of the need and the means of “performing their identities” seemed to have been in more advantaged positions than those who were not aware of these matters. Performance of class, nativity in Finland, and the ability to use one’s native language when accessing care allowed for significantly faster access to treatment as compared to informants lacking these privileges. In addition, non-binary informants articulated a specific need to perform their gender along the binary —and therefore lie— when presenting themselves at the trans clinics, in order to get the desired treatments, they are hoping for. It is clear from the analyzed materials that the intersectional aspects of individual identities contribute to the making of structural inequalities in gender-affirming healthcare in Finland.

The findings of the study are very much in line with those of previous international research about the experiences of transgender individuals when accessing gender-affirming care [7, 13, 39]. When locating the study in the broader international context, it confirms that there are a number of changes that are to be implemented in order to decrease structural inequalities in the gender-affirming health service system in general, but also on the broader societal level in particular. Based on the results of this study, such changes include for example, consciously educating interpreters and translators about questions relating to sexual and gender diversity, providing translated forms for healthcare services in a more diverse set of languages, and educating health and social service professionally on questions pertaining to cultural, sexual and gender diversity. Furthermore, allowing the possibility to provide gender-affirming services to asylum seekers should be explored further.

In order to develop these systems, and to contribute to reducing the stigma and inequalities transgender individuals of diverse backgrounds face both in the Finnish and in other societies, further investigation and research is needed on the subject matter, for example on the experiences of those who live in the more rural areas of Finland. Moreover, further research is needed on the healthcare experiences of gender minorities in Finland both within and outside the scope of transgender-specific healthcare, to reveal further structural inequalities in the existing systems.

## Data Availability

The data set associated with the paper is archived at the Finnish Institute of Health and Welfare (THL). The datasets analyzed during the current study are not publicly available due to ethical considerations. Data are however available from the authors upon reasonable request and with the permission of THL.

## Footnotes

1 Fin*. Laki transseksuaalin sukupuolen vahvistamisesta*; Eng. *Act on the legal recognition of gender of transexuals*.

2 Whilst recognizing these issues concerning the current legislation, a recent governmental proposal (STM009:00/2021) was created to tackle, re-evaluate and change the problematic parts of the current legislation.

3 International Classification of Diseases.

4 Whereas in the ICD-10 guidelines) the two currently used diagnostic codes related to gender affirmation are placed under “Mental and Behavioral Disorders,” in the ICD-11 guidelines, the diagnostic code “Gender incongruence” was introduced and placed under “Conditions related to sexual health” [4].

5 Swedish is also an official language in Finland, the number of native speakers however is 5,2% of the overall population – as of 2020 [36]. However, gender affirmation care in Swedish is often criticized, due to the lack of Swedish skills of the health professionals.

6 For further information on quota refugees in Finland, see: https://migri.fi/en/quota-refugees.

7 Various non-governmental organizations provide information, activities, and support-groups for queer individuals, however, informants suggested that the English language materials are not vast. Helsinki Pride Community runs a project in which they offer activities and organize peer-support for queer refugees and asylum-seekers. In addition, the non-governmental organization Loisto Settlement provides similar activities for victims of honor-related violence, many of them who are queer.

8 *Miyake* refers to Issey Miyake is a Japanese fashion designer, known for his luxury clothing designs, exhibitions, and fragrances.
